# Identification of Barramundi (*Lates calcarifer*) DC-SCRIPT, a Specific Molecular Marker for Dendritic Cells in Fish

**DOI:** 10.1371/journal.pone.0132687

**Published:** 2015-07-14

**Authors:** Emmanuelle Zoccola, Jérôme Delamare-Deboutteville, Andrew C. Barnes

**Affiliations:** The University of Queensland, School of Biological Sciences and Centre for Marine Science, Brisbane, Queensland, 4072, Australia; Temasek Life Sciences Laboratory, SINGAPORE

## Abstract

Antigen presentation is a critical step bridging innate immune recognition and specific immune memory. In mammals, the process is orchestrated by dendritic cells (DCs) in the lymphatic system, which initiate clonal proliferation of antigen-specific lymphocytes. However, fish lack a classical lymphatic system and there are currently no cellular markers for DCs in fish, thus antigen-presentation in fish is poorly understood. Recently, antigen-presenting cells similar in structure and function to mammalian DCs were identified in various fish, including rainbow trout (*Oncorhynchus mykiss*) and zebrafish (*Danio rerio*). The present study aimed to identify a potential molecular marker for DCs in fish and therefore targeted DC-SCRIPT, a well-conserved zinc finger protein that is preferentially expressed in all sub-types of human DCs. Putative dendritic cells were obtained in culture by maturation of spleen and pronephros-derived monocytes. DC-SCRIPT was identified in barramundi by homology using RACE PCR and genome walking. Specific expression of DC-SCRIPT was detected in barramundi cells by Stellaris mRNA FISH, in combination with MHCII expression when exposed to bacterial derived peptidoglycan, suggesting the presence of DCs in *L*. *calcarifer*. Moreover, morphological identification was achieved by light microscopy of cytospins prepared from these cultures. The cultured cells were morphologically similar to mammalian and trout DCs. Migration assays determined that these cells have the ability to move towards pathogens and pathogen associated molecular patterns, with a preference for peptidoglycans over lipopolysaccharides. The cells were also strongly phagocytic, engulfing bacteria and rapidly breaking them down. Barramundi DCs induced significant proliferation of responder populations of T-lymphocytes, supporting their role as antigen presenting cells. DC-SCRIPT expression in head kidney was higher 6 and 24 h following intraperitoneal challenge with peptidoglycan and lipopolysaccharide and declined after 3 days relative to PBS-injected controls. Relative expression was also lower in the spleen at 3 days post challenge but increased again at 7 days. As DC-SCRIPT is a constitutively expressed nuclear receptor, independent of immune activation, this may indicate initial migration of immature DCs from head kidney and spleen to the injection site, followed by return to the spleen for maturation and antigen presentation. DC-SCRIPT may be a valuable tool in the investigation of antigen presentation in fish and facilitate optimisation of vaccines and adjuvants for aquaculture.

## Introduction

Jawed fish are the earliest vertebrate order possessing both innate and adaptive immunity [1, 2]. Adaptive immunity is characterised by the development of an immune memory of previously encountered pathogens that allows a fast and specific response when reinfection by the same pathogen occurs. Adaptive immunity involves antigen recognition by antigen presenting cells (APCs) and an associated antibody response and pathogen elimination, generally performed by primed B and T-lymphocytes [3]. B-lymphocytes are specialised in producing antibodies that label pathogens and make them easier to identify by phagocytic cells [3]. T-lymphocytes assist in leucocyte activation or they destroy tumorous and virus infected cells, depending on the cell type [3]. T-cell activation is initiated by and is dependent upon the major histocompatibility complex (MHC) receptors which bind foreign and local peptides produced by protein degradation [4]. Naïve T-cells are activated after they are presented with foreign antigens that are bound on the MHC [4]. “Non-professional” APCs can be any cell in the organism and can only activate cytotoxic T-cells by displaying antigenic structures on MHC class I receptors or via stimulation by cytokines [5]. In contrast, “professional” APCs are specialised in priming naïve T-cells and can activate both cytotoxic and helper T-cells by displaying antigenic structures on MHC class I and II receptors respectively and by producing co-stimulatory molecules [6]. B-cell proliferation can also be indirectly activated by professional APCs [3]. The principal professional APCs in mammals are dendritic cells (DC), and are defined by their extended ability to engulf pathogens, present antigens and activate potent helper T-cells [7–9].

The existence of DC-like cells in teleosts has been controversial for many years, in part due to the absence of specific identification tools such as cell surface receptor markers, and due to significant differences between the immune systems of teleosts and mammals. For example, the lymph nodes and the bone marrow, which are essential to mammalian immunity by seeding the lymphoid organs, are absent in fish [1, 10]. Moreover, the lymph nodes are also the major site for antigen presentation, thus the main site where DCs interact with T-cells in mammals [11], and their absence in fish is an important consideration in the study of fish acquired immunity. Consequently, processes such as antigen presentation are still poorly understood in fish, and the lack of specific cell markers makes the study of these immune processes even more challenging. In spite of these challenges, dendritic-like cells have been identified recently in head kidneys (HK) and/or spleens of zebrafish (*Danio rerio*), Atlantic salmon (*Salmo salar*) and rainbow trout (*Oncorhynchus mykiss*) [12–15]. Organs such as the HK and the spleen in fish seem to effectively replace the missing leucocyte-producing mammalian organs. The HK has a major role in immune-endocrine interactions and, in fish, supplants the bone marrow as the main hematopoietic organ [1]. Fish spleen is analogous to mammalian spleen and is a likely site for T-cell-APC interactions [15]. However, the lack of DC-specific markers in fish means that the cells require laborious functional and morphological characterisation making the study of antigen presentation in fish extremely difficult. Recently, a zinc-finger protein, DC-SCRIPT, which is preferentially expressed in all subsets of dendritic cells in humans [16] and in mice [17], was discovered. Importantly, DC-SCRIPT was found to be identical to *Homo sapiens* ZNF366, which is homologous to the teleost fish *Fugu rubripes* fZnf1 gene [16]. This, in conjunction with the lack of confinement of DC-SCRIPT to a particular subset of DCs, makes it a logical target as a first molecular marker for fish DCs.

In the present study we identify DC-SCRIPT for the first time in a Perciform fish, the barramundi, *Lates calcarifer* and show specific expression in barramundi dendritic-like cells (bDCs). The order Perciformes represents the largest extant group of vertebrates (comprising 40% of known fish species) and includes an increasing proportion of the globally expanding aquaculture industry, particularly high value fish such as tilapias, snappers, sea bass, breams, jacks and barramundi, yet little work has been conducted to date on immunity in this order. Barramundi represent a good candidate for further research on perciform immunity as hatchery technology is well established, they are easy to keep under controlled conditions in recirculating systems and are of significant value to aquaculture throughout south east Asia [18]. Improved tools for *in vitro* models of the cellular processes leading to specific adaptive immunity are therefore significant in the refinement of vaccines for the aquaculture industry and our understanding of antigen presentation in lower vertebrate orders.

## Materials and Methods

### Experimental animals and husbandry

Barramundi (*Lates calcarifer*) juveniles of approximately 30–100g were obtained from a commercial aquaculture facility near Cairns, Queensland, Australia. Fish from this farm are specific pathogen free fish as the direct supply of filtered bore water to the hatchery and farm coupled with strict biosecurity prevent exposure to common fish pathogens. Fish were transported to The University of Queensland by air freight and acclimatised for 2 weeks in a recirculating system in eight 84 L cylindrical food-grade plastic tanks with individual aeration, all connected to a 260 L sump equipped with a protein skimmer and a bio-filter. The water temperature and the salinity were maintained at 28 ± 2°C and 15 part per thousand (ppt) respectively. Water quality was checked daily for ammonia, nitrite, nitrate and pH, and water exchanges were applied as required. Fish were fed to satiation once daily with a commercial diet for barramundi (Ridley Aqua Feed).

#### Serum collection

Barramundi were bled aseptically from the caudal vein and the blood was allowed to clot for 1 h at room temperature. Samples were centrifuged at 12,100 x g for 10 min and the supernatant (serum) was stored at -20°C until further use. Prior to use, serum was heat inactivated (HI) for 30 min at 56°C.

### Identification of Barramundi DC-SCRIPT

#### DNA and RNA extraction

DNA was extracted from cultured barramundi HK and spleen cells (in 75 cm^3^ flasks, 10^7^ cells/mL) using Nucleospin Tissue DNA kit (Machery Nagel) according to the manufacturer’s instructions. Extracted DNA was subsequently used for the construction of four DNA-libraries as described in the Universal Genome Walker 2.0 kit (Clontech Laboratories, Inc). RNA was extracted from HK and spleen cultured cells (in 75 cm^3^ flasks, 10^7^ cells/mL) using the RNeasy Mini Kit (QIAGEN) according to the manufacturer’s instructions. Extracted RNA was subsequently synthesized into complementary DNA (cDNA) RACE libraries using the SMARTer RACE cDNA Amplification kit (Clontech Laboratories, Inc) section V.

#### Primer design, genome walking and 5’ 3’ RACE PCR

Initially, degenerate primers for teleost DC-SCRIPT were designed from conserved sequences derived from multiple alignments (CLUSTALW) of hypothetical DC-SCRIPT identified by nucleotide Basic Local Alignment Search Tool (BLAST; http://blast.ncbi.nlm.nih.gov/) of completed teleost genomes using human DC-SCRIPT (NM_152625.1) as the query sequence. Sequences included in the alignment were derived from the genomes of *Pundamilia nyererei* (XM_005741743), *Maylandia zebra* (XM_004547137), *Haplochromis burtoni* (XM_5919046), *Oreochromis niloticus* (XM_005452162), *Takifugu rubripes* (XM003974872) and *Danio rerio* (XM_693094.5). Amplicons derived from genomic DNA by degenerate PCR were sequenced and those with homology to DC-SCRIPT by BLAST were employed to design gene specific primers for genome walking and RACE in accordance with the manufacturer’s instructions (Clontech). New primers were designed following each round of walking or RACE to obtain sufficient sequence for further experimentation.

#### mRNA Fluorescence In Situ Hybridization (FISH) and immunocytochemistry

To identify bDCS in mixed and enriched cell cultures, mRNA FISH was employed. Around 500 base pairs of DC-SCRIPT cDNA sequence, downstream of the zinc finger region, was used to design mRNA custom FISH probes using the Stellaris FISH Probe Designer tool available online at www.biosearchtech.com/stellarisdesigner (Biosearch Technologies). In total, 32 FISH probes were designed and conjugated to CAL Fluor 590 (Biosearch Technologies). Mixed-cell populations were then allowed to adhere on round glass coverslips at 28°C, were subsequently stimulated for 2 h with peptidoglycan (PTG; 10 μg/ml), and underwent immunocytochemistry (ICC) fluorescent staining procedures. Different treatments included anti IgM (1:1000 primary antibody sheep IgG vs. barramundi IgM; 1 μg/mL secondary antibody donkey vs. sheep IgG conjugated with AlexaFluor 405 for combined staining or with AlexaFluor 594 for separate staining), anti MHCIIa (1:1000 primary antibody rabbit IgG vs. zebrafish MHCIIa (Sapphire Bioscience, Product LS-C210021); 1 μg/mL secondary antibody goat vs. rabbit IgG conjugated with AlexaFluor 488) and *in-situ* hybridisation with the DC-SCRIPT probes (50 ng), either combined or stained separately. Antibodies and Stellaris FISH probe titrations were previously performed to determine optimal concentrations. Controls consisted of the same procedure while omitting the primary antibodies or the Stellaris FISH probe. The adherent cells were hybridised and stained, with some modifications, following the Stellaris FISH probes manufacturers instructions (For simultaneous Stellaris FISH and immunofluorescence using adherent cells) available online at www.biosearchtech.com/stellarisprotocols. Briefly, the cells were fixed in 4% paraformaldehyde (Electron Microscopy Science) for 10 min at room temperature (RT), washed in 1X PBS (5 min; RT) and permeabilized with Triton-X for 3 min (Sigma; RT). The cells were then washed in 1X PBS twice (5 min; RT) and blocked in 5% donkey serum, 5% goat serum, 0.1% bovine serum albumin for 30 min at RT, followed by two more washes in 1X PBS (5 min; RT) and an incubation in the dark at 37°C for 4 h in hybridisation buffer containing 50 ng of FISH probe and primary antibodies at the dilutions specified above. Subsequently, the cells were washed three times in 1X PBS (5 min; RT) and incubated in wash buffer as recommended by the manufacturer (Stellaris FISH Probes protocol) containing the appropriate secondary antibodies (see above) for 30 minutes at 37°C in the dark. When stains were combined, the coverslips were mounted at this point using VECTASHIELD HardSet Mounting Medium (Vector Laboratories). When stains were separated, cell nuclei were stained with DAPI (Invitrogen; 2.5 μg/mL) for 15 minutes at RT in the dark before the coverslips were mounted using VECTASHIELD HardSet Mounting Medium (Vector Laboratories).

#### Fish injection and RNA extraction for qRT-PCR

Barramundi were injected in the peritoneal cavity with 100 μL of either sterile phosphate buffered saline (PBS control) or a mixture of lipopolysaccharide (LPS; 10 μg/mL) and peptidoglycan (PTG; 10 μg/mL) diluted in PBS. LPS and PTG were chosen based on both *in vivo* and *in vitro* induction of immune response in fish/fish cells previously reported [19, 20]. At 6 h, 24 h, 72 h and 7 d post-injection, four fish were sampled from both control and treatment groups. For each fish, the spleen and head-kidney were dissected aseptically and kept in RNAlater until further processing.

RNA was extracted using the RNeasy extraction kit (QIAGEN), according to the manufacturer’s instructions. Tissues kept in RNAlater were disrupted by serial passage through a 25-gauge needle mounted on a 1 mL syringe before processing. RNA was eluted in 40 μL of nuclease free water.

#### Quantitative real-time PCR for relative quantification of DC-SCRIPT expression

RNA samples were treated with DNAse (RNAse-free DNAse set, Qiagen) to remove any trace genomic DNA then converted to cDNA using the QuantiTect RT kit (QIAGEN) according to the manufacturer’s instructions. cDNA was quantified by Qubit Fluorimetry and 0.5 ng per sample was optimised as template to yield approx. 100% efficiency in 10 μL real-time PCR reactions with 0.2 picomoles of each primer pair specific for DC-SCRIPT and three endogenous reference genes (Table 1) [21]. Primers were designed to span the intron/exon boundaries to eliminate amplification of any traces of genomic DNA. Amplification of 3 technical replicates from each of the 4 biological replicate samples per treatment and control was performed in 384 well plates on a ViiA 7 Real-Time PCR system (Applied Biosystems) using SYBRgreen MasterMIx (Invitrogen) and cycling parameters as follows: 95°C for 10 min followed by 40 cycles of 95°C for 15 s, 54°C for 30 sec and 62°C for 30 sec, then a final melt curve at 95°C for 15 s, 60°C for 1 min and 95°C for 15 s. All temperature cycling was performed with acceleration at 1.6°C/s. The PCR exponential amplifications of each gene were calculated based on the slope estimated by the Cq value of a serial dilution in a preliminary validation experiment against Log_10_ of the cDNA amount per reaction, described by E = 10^-1/slope^ [22]. Any variation in amplification efficiency was then accounted for during normalisation of relative gene expression differences between the treatment samples and the control samples. The normalisation factor was calculated based on the geometric mean of the relative quantities of three reference genes using REST [23, 24]. Cq data were analysed with REST based on a pairwise fixed reallocation randomisation test [24], in which the normalised relative quantities were calculated based on the ratio of the group means for the target gene against the normalised reference genes. The result represented the up- or down-regulation of the treatment group (i.e. LPS/PTG-injected) compared to the control group (i.e. PBS injected).

**Table 1:** Primers employed for qRT-PCR. DC-SCRIPT primers were designed in this study. Endogenous reference gene primers are as reported previously [21].

Gene	Abbreviation	Primer pair (RT-qPCR)
Elongation factor-1 α	ef1-α	F:AAATTGGCGGTATTGGAAC R:GGGAGCAAAGGTGACGAC
18S ribosomial RNA	18S	F:CGCCTGAATACCGCAGCTAG R:AGAACGGCCATGCACCACCAC
α-tubulin	α-tub	F:GGCACTACACAATCGGCAAAGAGA R:TCAGCAGGGAGGTAAAGCCAGAGC
Dendritic cell specific transcript	DC-SCRIPT	F:AACAGCACACGCTCACTCAC R:CGATCATGTGAGCCTTGAGA

### Cell culture & functional assays

#### Cell culture from hematopoietic organs

Barramundi were killed by overdose of anaesthetic (Aqui-S). Fish were bled from the caudal vein before the spleen and HK were removed aseptically. The organs were placed on ice in complete L-15 medium: L-15 Leibovitz medium with phenol red (Invitrogen) supplemented with HI 10% foetal bovine serum (FBS), 2% Nystatin (N; 10,000U/mL stock) and 1% penicillin-streptomycin (PS; 10,000U/mL stock). The organs were then gently pushed through a 100 μm cell strainer with the plunger of a 1 mL tuberculin syringe to obtain a single cell suspension in supplemented medium. Cells were counted and adjusted to a concentration of 1 x 10^7^ cells/mL in supplemented L-15 and plated in 25 cm^2^ or 75 cm^2^ culture flasks. Cells were incubated at 28°C for 5–14 days. During this period, non-adherent cells were harvested and the removed medium was replaced by fresh supplemented medium.

#### Barramundi DCs harvesting and enrichment

Prior to harvest, culture flasks were gently agitated and medium containing the suspended non-adherent cells was collected. The suspension was layered over a discontinuous Percoll gradient (Sigma) at d = 1.058 g/mL and d = 1.048 g/mL and centrifuged for 30 min at 800 x g with no brake at 23°C, as adapted and modified from Bassity and Clark (15). The cells at the interface between the Percoll layers were collected and washed at 23°C in fresh medium (10 min, 400 x g) before further use.

#### Transwell migration assay

For this study, two isolates of *Streptococcus iniae* (strains QMA0076 and QMA0248) from disease outbreaks in Australian barramundi farms (in Queensland and New South Wales respectively) were chosen. These strains had previously been employed in a vaccination trial in which experimental fish responded poorly to vaccines prepared from QMA0076, but responded strongly with consistently high antibody response to vaccines prepared with QMA0248 [25]. The migratory ability of bDCs was assessed using a 12-well Transwell culture plate (Corning) with membranes presenting 3 μm pores, which allowed active migration between upper and lower compartments, but were substantially smaller than the cells (10–20 μm) to minimise passive transfer between the chambers. For each treatment, 1.5 mL of 0.5% FBS/L-15 was placed into the lower part of the Transwell and either lipopolysaccharide (LPS; 5 μg/mL), peptidoglycan (PTG; 10 μg/mL) or live *S*. *iniae* (strains QMA0076 or QMA0248 at a multiplicity of infection (MOI) of 0.1 and 1) were added to the medium. The control was composed of medium alone in the lower chamber. Barramundi DCs (500 μL of 4 x 10^5^ cells/mL), resuspended in 0.5% FBS/L-15, were added to the upper chamber. The Transwell plate was then incubated for 4 h at 28°C to allow migration. After 4 h, the cells remaining in the upper chamber were removed by gently swabbing a cotton bud against the membrane. The cells attached on the lower part of the membrane were detached with trypsin (Sigma; 10 μg/mL) for 1.5 min. Cells were then stained with DAPI (Invitrogen; 2.5 μg/mL for 15 min) and counted on a haemocytometer using fluorescent microscopy.

#### Phagocytosis assay

The preliminary assessment of bDCs phagocytic capacity was performed in 24-well plates using fluorescent latex beads. The bead stock suspension (Fluoresbrite Yellow Green microspheres 0.2 μm, ~5.68 x 10^12^ particles/mL, 2.65% latex, Polysciences, Inc.) was diluted 50-fold in phosphate buffered saline (PBS) pH 7.4 and opsonised by addition of HI barramundi serum to 10% (v/v). Barramundi DCs concentration was adjusted to 5 x 10^6^ cells per well in 500 μL L-15 in a 24-well tissue culture plate. Then opsonised beads were added to each well, at a 1:10 dilution in 10% FBS/L-15 and plates centrifuged at 500 x g, 23°C for 5 min to bring the beads and cells into contact, as adapted and modified from Bassity and Clark (15). After 2 h incubation at 28°C, the plates were put on ice to stop the cell activity and were observed by microscopy directly in culture or as cytospins (see below).

Further analysis of bDCs phagocytic capacities was performed using flow cytometry. When fluorescent beads were used, the protocol remained the same as described above, with the following exceptions: After incubation (2 h at 28°C), the cells were washed from the plates in medium and transferred to 5 mL cytometry tubes before being put on ice to stop the cell activity until flow cytometry analysis.

For phagocytosis of bacterial strains, *S*. *iniae* QMA0076 and QMA0248 were adjusted to an optical density at 600nm (OD_600_) of 1 (~10^8^ bacteria/mL). They were then stained with 1μM BacLight Green (BLG)(Life technologies) for 15 min at RT before being washed extensively in PBS and diluted 100-fold in 10% FBS/L-15. Barramundi DCs concentration was adjusted to 5 x 10^6^ cells per well in 500 μL and the medium containing stained bacteria was added resulting in a final multiplicity of infection (MOI) of 1.0. After incubation for 2 h at 28°C, the cells were washed from the plates in medium, transferred to 5 mL flow cytometry tubes and put on ice to stop the cell activity until flow cytometry analysis.

#### T lymphocyte isolation by E-rosette

After sterile removal of the spleen from euthanased barramundi, a single cell suspension was obtained by forcing the spleen through a 100 μm cell strainer with the plunger from a 1 mL tuberculin syringe. The suspension was then centrifuged on a d = 1.072 g/mL and d = 1.050 g/mL discontinuous Percoll gradient at 800 x g, 23°C for 30 min, as adapted and modified from Tumbol, Baiano [26]. The leucocytes, located at the interface between the two Percoll layers, were collected and diluted 2-fold in L-15 medium. Cells were washed by centrifugation at 400 x g, 23°C for 10 min. After centrifugation, the supernatant was discarded and the cell pellet resuspended in L-15 medium supplemented with 10% FBS/1% PS.

To obtain a pure culture of live T-cells to test in the proliferation assay, the E-rosette method was used, as adapted and modified from Madsen, Johnsen (27). Sheep red blood cells (SRBC) in Alsever’s solution were diluted 1:1 in complete L-15 medium. Equal volumes of spleen leucocytes and SRBC in L-15 were then mixed in a 1.5 mL Eppendorf tube and centrifuged for 5 min at 5870 x g to bring the leucocytes and SRBC in contact. The tubes were then incubated at 28°C overnight to allow formation of rosettes (S1 Fig). On the next day, the cells were resuspended and layered and centrifuged over a d = 1.072 g/mL and d = 1.050 g/mL discontinuous Percoll gradient at 800 x g, 23°C for 30 min. The supernatant was discarded and the pellet containing the SRBC and the T-cells was washed twice in PBS. RBC lysis buffer (1 mL) was then added to the pellet and, when the suspension became translucent, complete L-15 medium was added to stop the reaction. The enriched T-cells, released from the rosettes, were then washed twice and resuspended in L-15/10% FBS medium.

#### T-cell proliferation assay

Responder cells (T-cells) and stimulator cells (bDCs) from different fish were counted and resuspended in L-15 supplemented with 5% barramundi serum, 1% PS at a concentration of 5 x 10^5^ cells/mL. Responder cells were stained using 5 μM/mL carboxyfluorescein succinimidyl ester (CFSE; Sigma) for 30 min at 28°C. Cells were then washed twice in PBS by centrifugation. T-cells and bDCs were mixed together at a 1:1, 1:2, 1:4 and 1:8 stimulator to responder ratio and incubated for three days (experimentally determined to be optimal) at 28°C in the dark. Negative controls were composed of responder cells alone. On day 3, cells were analysed by flow cytometry using decrease in CFSE fluorescence as an indicator of proliferation.

### Flow cytometry

Flow cytometry data were obtained using the BD FACSAria II (BD Bioscience). Data were analysed using the DIVA software. Hoechst and propidium iodine (PI) fluorescent dyes (both from Life Technologies) were used according to the manufacturer’s instructions to identify and exclude debris and dead cells respectively. The FITC voltages for the phagocytosis and proliferation functional assays were set at 419 V and 520 V respectively and the 605/12 violet voltage was set at 485 V for the Stellaris FISH probe labelling.

### Microscopy and non-fluorescent staining

Cultures were viewed with an Olympus CKX41 inverted microscope and cytospin slides were viewed using an Olympus BX41 epifluorescent microscope. On both microscopes, images were captured with an Olympus DP26/U-CMAD3 camera and optimised with the imaging software CellSens (Olympus Optical Co. Ltd, Japan). Cytospins were performed with the Cellspin I (Tharmac GmbH), stained with the Hemacolor staining kit (Merck Millipore), according to manufacturer instructions, and subsequently mounted with Permount medium (Fisher Scientific). Cytospins of cells previously stained with fluorochromes were mounted using DABCO anti-fading medium (Sigma).

### Statistical analysis

Data analysis was performed with R v2.15.0 (R Core Team, 2012). Before analysis, homogeneity of variance was checked using Cochran’s test. Data from the qRT-PCR and the migration assay were analysed using analysis of variance (ANOVA) tests. Where ANOVA indicated significant differences, it was followed by pairwise comparison t-tests. As variances were not homogeneous for the phagocytosis assay, the non-parametric Mann-Whitney U test was used.

### Ethics statement

All animal work was conducted in accordance with Animal Care and Protection Act and the NHMRC Code of Practice. Work was conducted under the University of Queensland Animal Ethics Committee Approval No. SBS/056/13/ARC “Cellular immunity in fish: Robust defence against infection or Achilles heel?”

## Results

### DC-SCRIPT is a potential marker for DCs in barramundi

**Fig 1:**
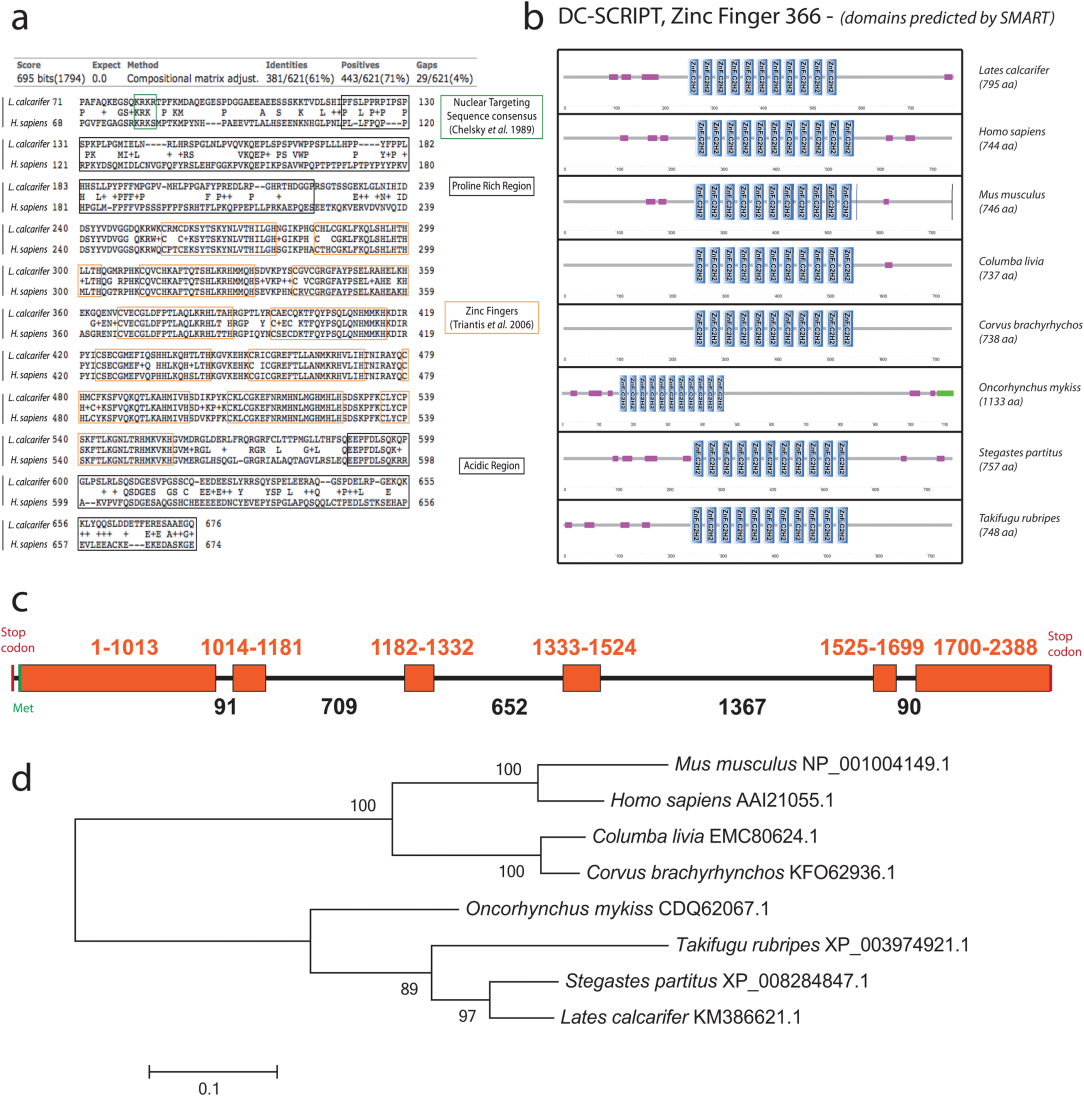
Amino-acid sequence alignment, domain organisation, gene organisation and phylogeny of DC-SCRIPT from *L*. *calcarifer*. (a) Amino acid sequences comparison between barramundi (*L*. *calcarifer*) and human (*H*. *sapiens*) DC-SCRIPT, aligned on the NCBI website (http://www.ncbi.nlm.nih.gov) by protein BLAST. The green box represents the nuclear targeting sequence consensus (KRK*) as per Chelsky, Ralph [51]. The black boxes represent a proline rich region and an acidic region at the N- and C-ends respectively. The 11 orange boxes represent the 11 zinc finger sequences as identified by Triantis, Trancikova [16]. (b) Domain organisation of DC-SCRIPT from different species predicted using SMART online software. Blue rectangles represent C2H2 zinc fingers, pink rectangles represent low complexity regions and the green rectangle represents a coiled coil region. (c) Gene organisation of barramundi DC-SCRIPT. The orange boxes represent the exons. Numbers are representative of the number of base pairs, totalling 5337 bp between stop codons. (d) Phylogenetic tree is representative of a Maximum Likelihood analysis performed on CLUSTALW aligned sequences (MEGA 5.2.2).

A putative gene for DC-SCRIPT was identified in barramundi genomic DNA and in cDNA derived from RNA extracted from stimulated enriched dendritic cells, by genome walking and RACE PCR respectively. A complete cDNA was obtained comprising 2385 nucleotides (nt) translating to a 795 amino acid (aa) putative protein with 11 conserved C_2_H_2_-like zinc fingers, a proline rich region at the N-terminal and an acidic region at the carboxy terminal end (Fig 1a). Nucleic acid binding and metal ion binding functions were identified with InterProScan [28]. Domain organisation was predicted using SMART online software (http://smart.embl-heidelberg.de) and was compared to a panel of DC-SCRIPT/Zinc-finger protein 366 from mammalian, aviary and fish species (Fig 1b). Comparison of RACE-derived cDNA sequence with gDNA obtained by genome walking revealed that Barramundi DC-SCRIPT comprises 6 exons (Fig 1c). Complete cDNA and gDNA sequences have been deposited in Genbank with accession number KM386621. The cDNA sequence and the translated protein sequence were highly homologous to predicted zinc-finger protein 366 from multiple teleost and bird hosts (including damselfish (*Stegastes partitus*), puffer fish (*Taifugu rubripes*), rock dove (*Columba livia*) and American crow (*Corvus brachyrhynchos*)) and to DC-SCRIPT from human (*Homo sapiens*) and mouse (*Mus musculus*) using BLAST (http://blast.ncbi.nlm.nih.gov/). The close homology was supported by phylogenetic analysis using maximum likelihood (MEGA 5.2.2 for Macintosh, Fig 1d).

**Fig 2:**
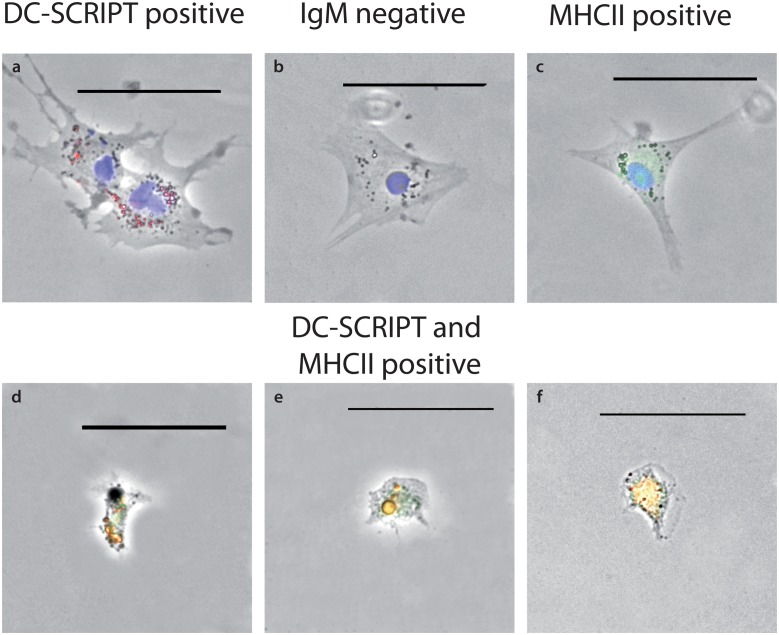
Antibody labelling of putative DCs. Antibody and Stellaris DC-SCRIPT FISH probe labelling of putative DCs: (a) DC-SCRIPT FISH probe in red and DAPI (nuclear stain) in blue, (b) DAPI (nuclear stain) in blue, (c) MHCII antibody in green and DAPI (nuclear stain) in blue, (d-f) DC-SCRIPT FISH probe in red, MHCII antibody in green. Scale bars represent 50 μm.

To determine specificity of DC-SCRIPT to barramundi DCs, mRNA FISH was used, employing 32 CAL Fluor 590 labelled probes downstream of the zinc finger motif to eliminate possible non-specific hybridisation to other zinc-finger containing transcription factors. Cells exhibiting dendrite-like protrusions were positively labelled with Stellaris DC-SCRIPT FISH probes as well as with anti-MHCII antibody but were not labelled with anti-IgM antibody (Fig 2d). Smaller, rounded cells were stained with both anti-MHCII and anti-IgM antibodies, but not with Stellaris DC-SCRIPT FISH probes, indicative of probable B-cells (S2 Fig). Macrophages appeared as irregularly shaped cells with regular nuclei and stained positively with anti-MHCII antibodies, but were negative for DC-SCRIPT and IgM (data not shown).

**Fig 3:**
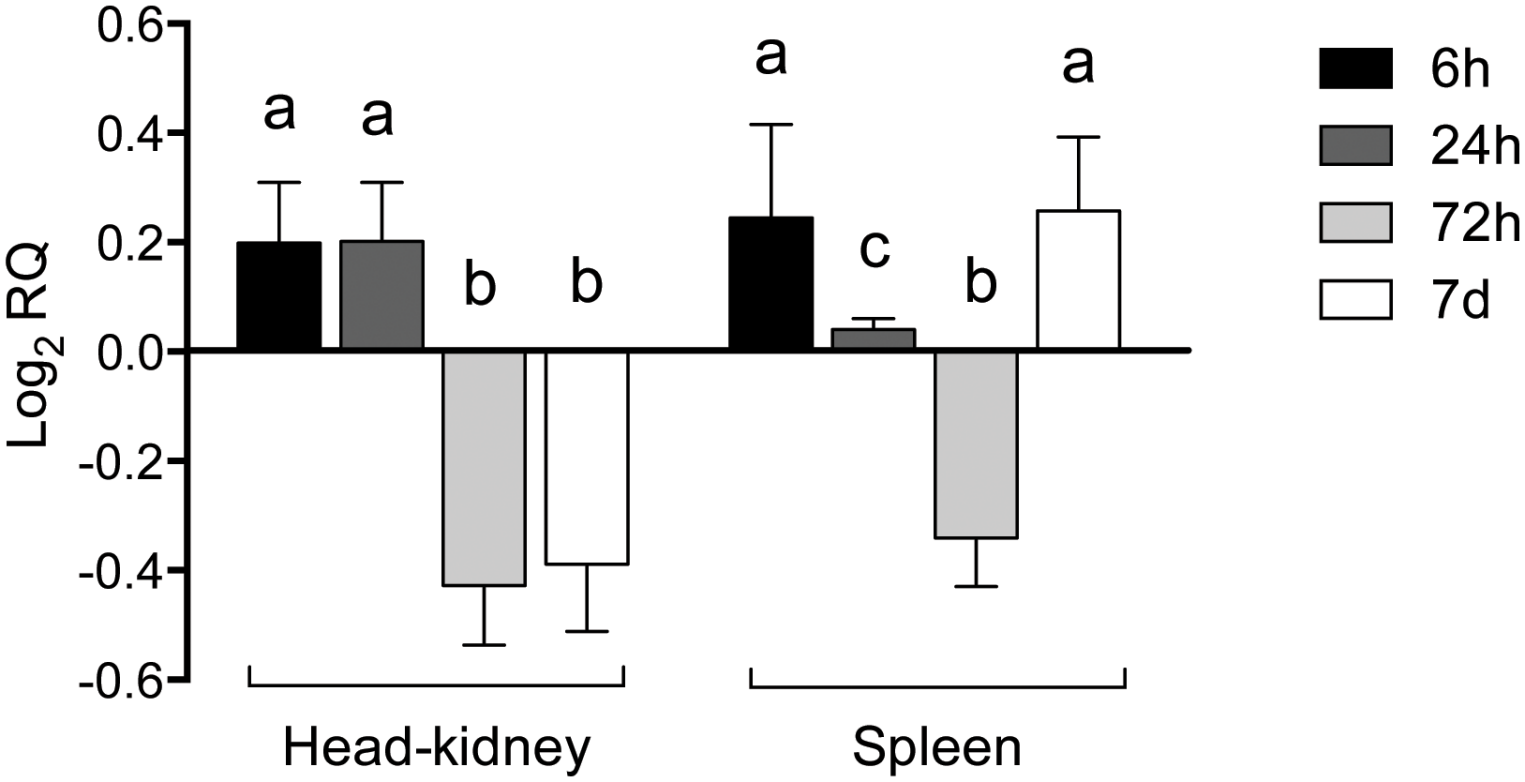
DC-SCRIPT expression in barramundi spleen and HK following a time course post-injection with LPS and PTG. Different letters represent statistically significant differences (*p* <0.05). Data are derived from 4 biological and 3 technical replicates per treatment/control.

To investigate DC-SCRIPT expression in haematopoietic tissues in fish, head kidney and spleen samples were analysed at 4 times between 6 h and 7 days post-injection with PTG and LPS. DC-SCRIPT expression was higher in both spleen and HK at 6 h post injection compared to PBS injected controls. However, after 24 h, DC-SCRIPT expression decreased in spleen but remained elevated in HK relative to controls. After 72 h, relative expression of DC-SCRIPT was lower in both spleen and HK and finally, after 7 d, DC-SCRIPT expression was once again higher in spleen but remained low in HK relative to PBS injected controls (Fig 3).

### Morphological identification

#### Identification of dendritic-like cells in hematopoietic cell cultures

**Fig 4:**
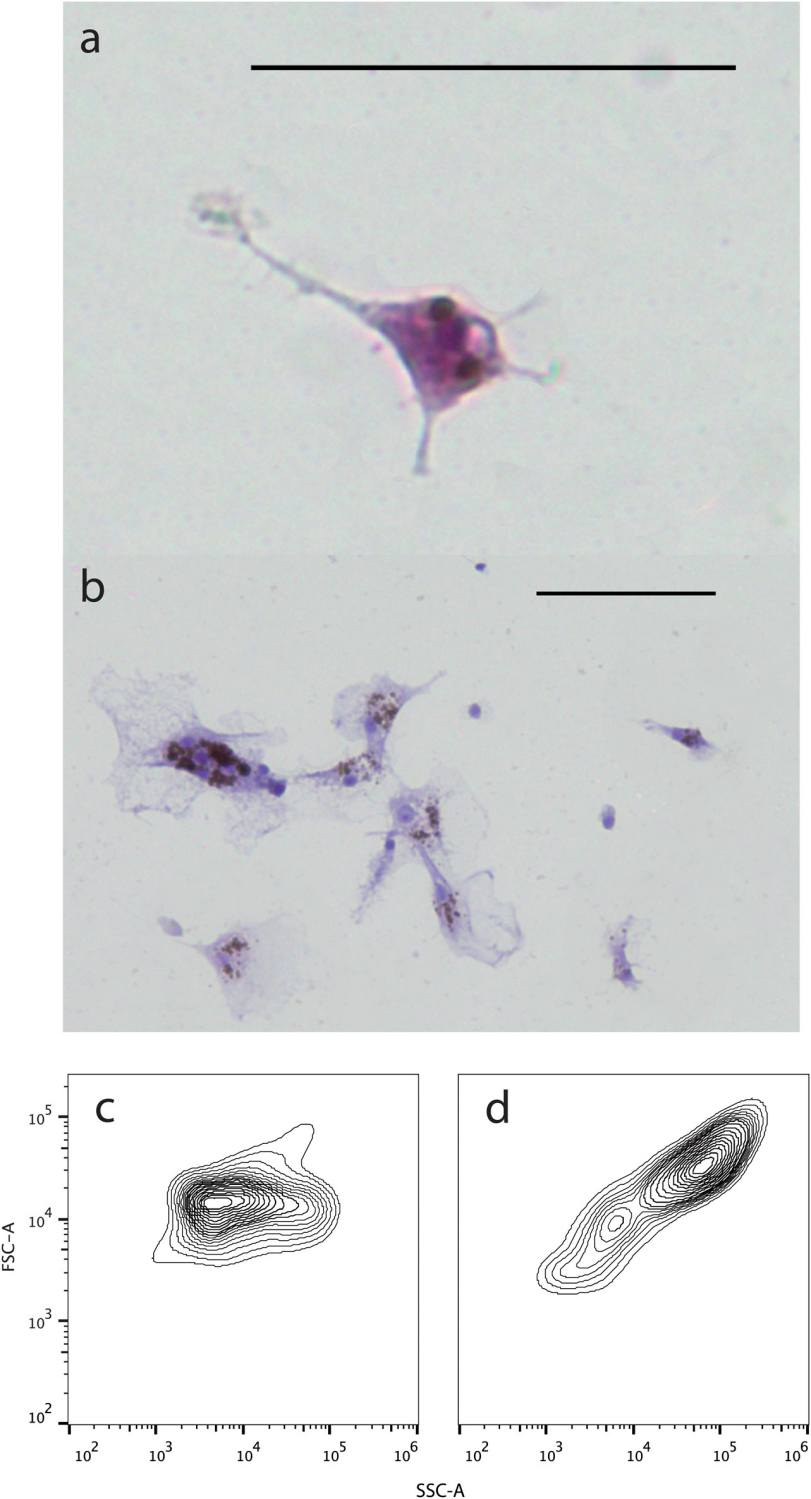
Barramundi DC morphology and isopycnic enrichment. (a-b) Dendritic-like cells isolated from *L*. *calcarifer* and stained with Hemacolour. (c) Forward and side scatter of barramundi spleen/HK cell populations before isopycnic enrichment and (d) after isopycnic enrichment.

The morphological traits used to identify bDCs were the presence of dendrites on the cells’ surface and their irregular nuclei, based on Steinman, Kaplan (29), Lu, Hsieh (30), Ganassin and Bols (31) and Bassity and Clark (15) (Fig 4a and 4b). After overnight incubation of HK and spleen-derived cultures, many cells, comprising large poly-nucleated melano-macrophages and macrophages, had adhered and formed clumps. Non-adherent cells were composed of small, round cells suggestive of lymphocytes/thrombocytes and larger, round, monocyte-like cells. On further sub-culture, these monocyte-like cells acquired an irregular shape with a branched morphology. Isopycnic separation on a 1.058 g/mL continuous Percoll gradient resulted in enrichment of the population in favour of larger, more granular cells with a low buoyant density, similar to mammalian DCs (mDCs) [29]. Enrichment was substantial, with an increase in putative bDCs from 2.07% to 63.02% of the population (determined by flow cytometry, Fig 4c and 4d).

### Functional identification

#### Barramundi DCs are migratory and phagocytic cells

**Fig 5:**
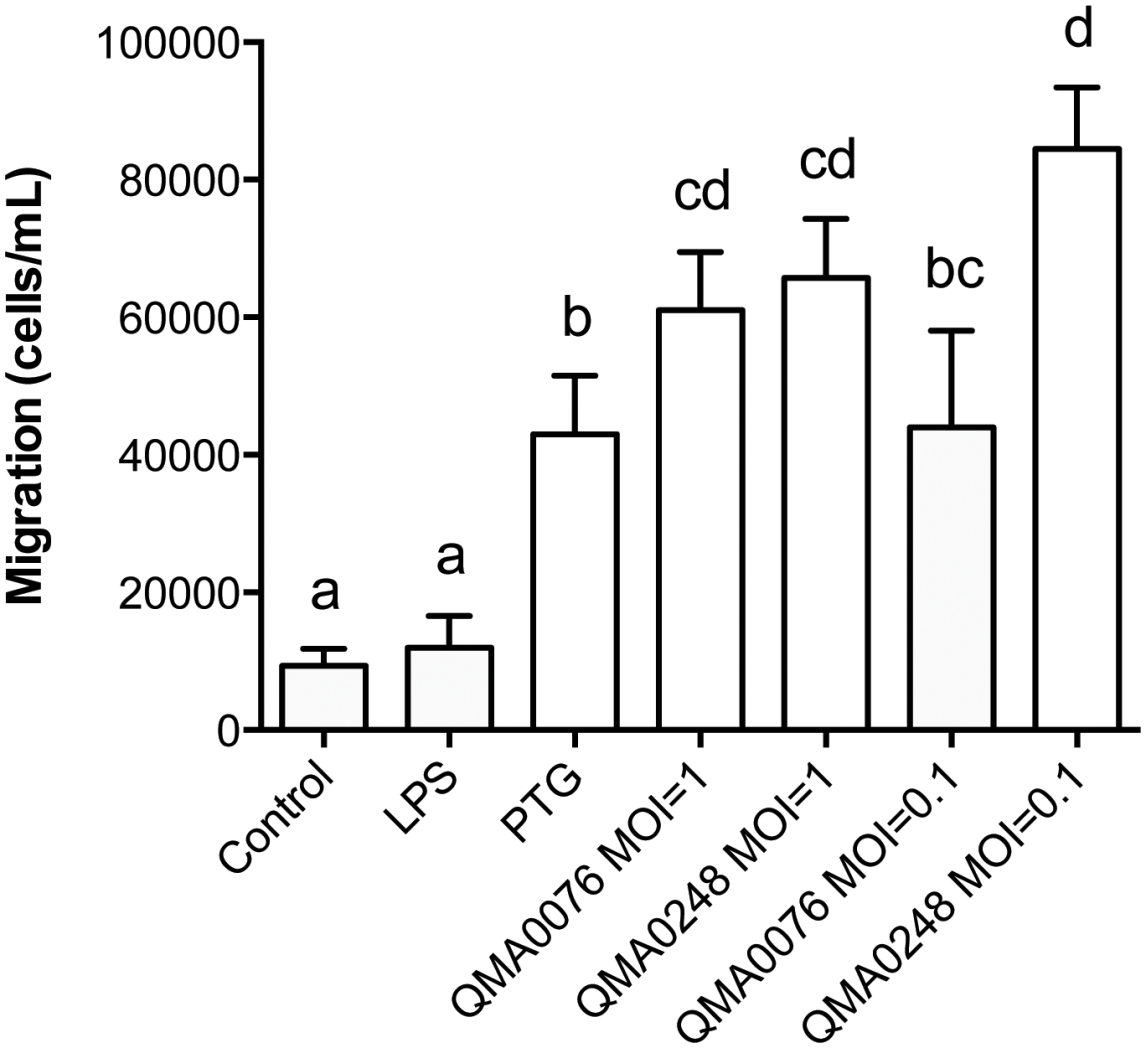
Barramundi DCs migration towards different chemical cues. Different letters represent statistically significant differences. Barramundi DCs migrated significantly towards all cues (*p* < 0.001 for PTG, QMA0076 and QMA028 at MOI 1 and QMA0248 at MOI 0.1; *p* < 0.01 for QMA0076 at MOI 0.1) with the exception of LPS, which bDCs did not migrate towards significantly (*p* = 0.076).

Trans-membrane migration and flow cytometry were used to assess chemotactic migration and phagocytosis capacity of bDCs. When exposed to Toll-like receptor (TLR) ligands and bacteria in the migration assay, bDCs actively migrated through the pores in the membrane towards PTG (TLR-2) and both *S*. *iniae* strains (*p* < 0.01) but not significantly towards LPS (TLR-4) (*p* = 0.076) (Fig 5). The migration was defined “active” because the cells, measuring approximately 10 μm, had to pass through 3 μm pores in order to reach the stimulants. Notably, bDCs migration towards either of *S*. *iniae* strains was not dose dependent, but at MOI 0.1 there was a significant difference in migration between the two isolates (*p* < 0.05, Fig 5).

**Fig 6:**
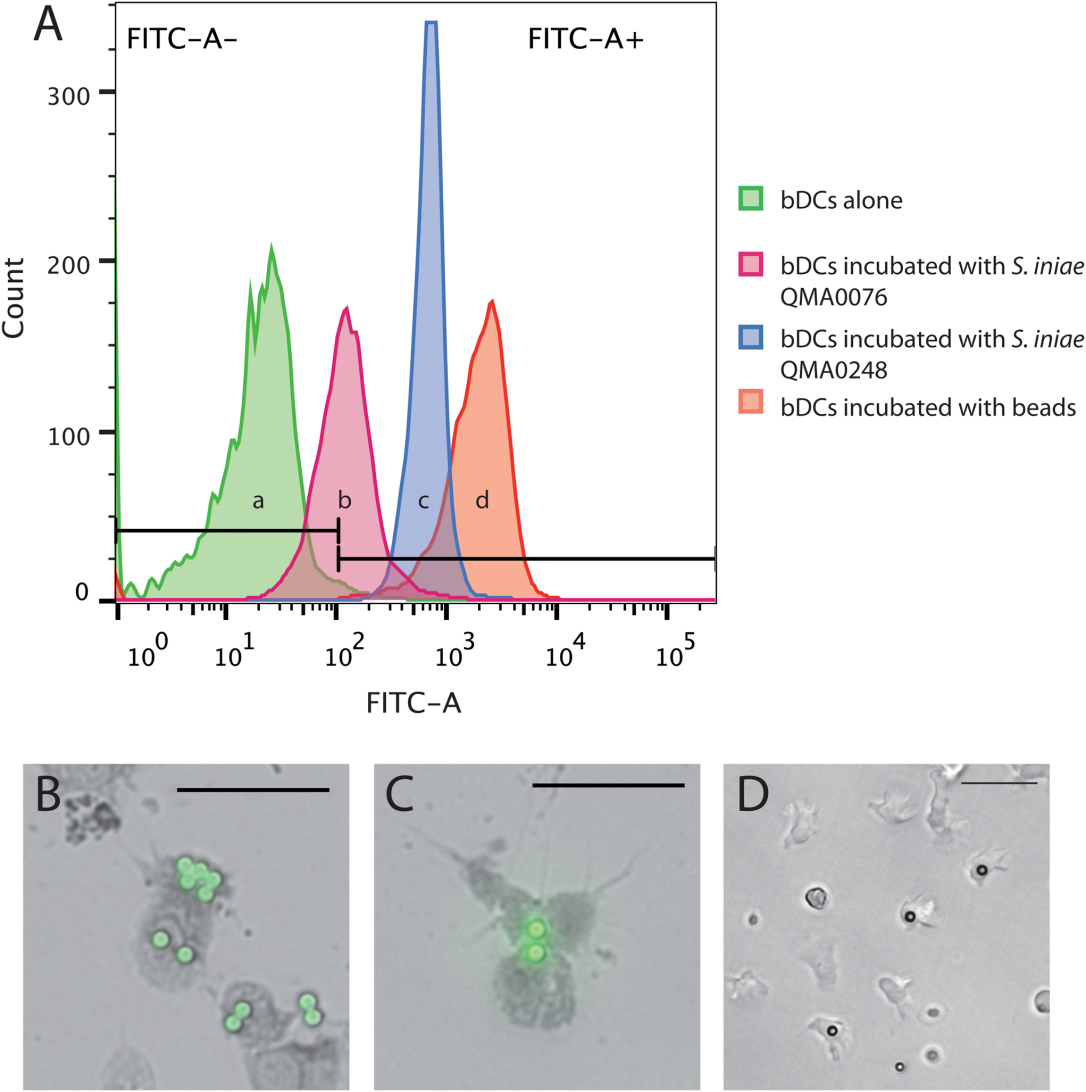
Phagocytic capability of bDCs. (A) (a) Histograms showing barramundi dendritic cells alone, in presence of stained bacteria (b) QMA0076 and (c) QMA0248 and (d) in presence of fluorescent beads. Note that ingestion of beads and bacterial cells shifts fluorescence of DCs fluorescence significantly the right along the x-axis (z > 1.96) in b, c and d when compared to a. (B-C) Fluorescent microscopy and (D) inverted light microscopy of dendritic-like cells incubated with fluorescent beads. Scale bars represent 50 μm.

When incubated with micro beads for 2 h, bDCs were able to ingest them, demonstrating their ability to phagocytose foreign particles (Fig 6). Dendritic cells were also able to ingest two strains of the barramundi primary bacterial pathogen *S*. *iniae* (Fig 6).

#### Barramundi DCs trigger the proliferation of responder T-cells

**Fig 7:**
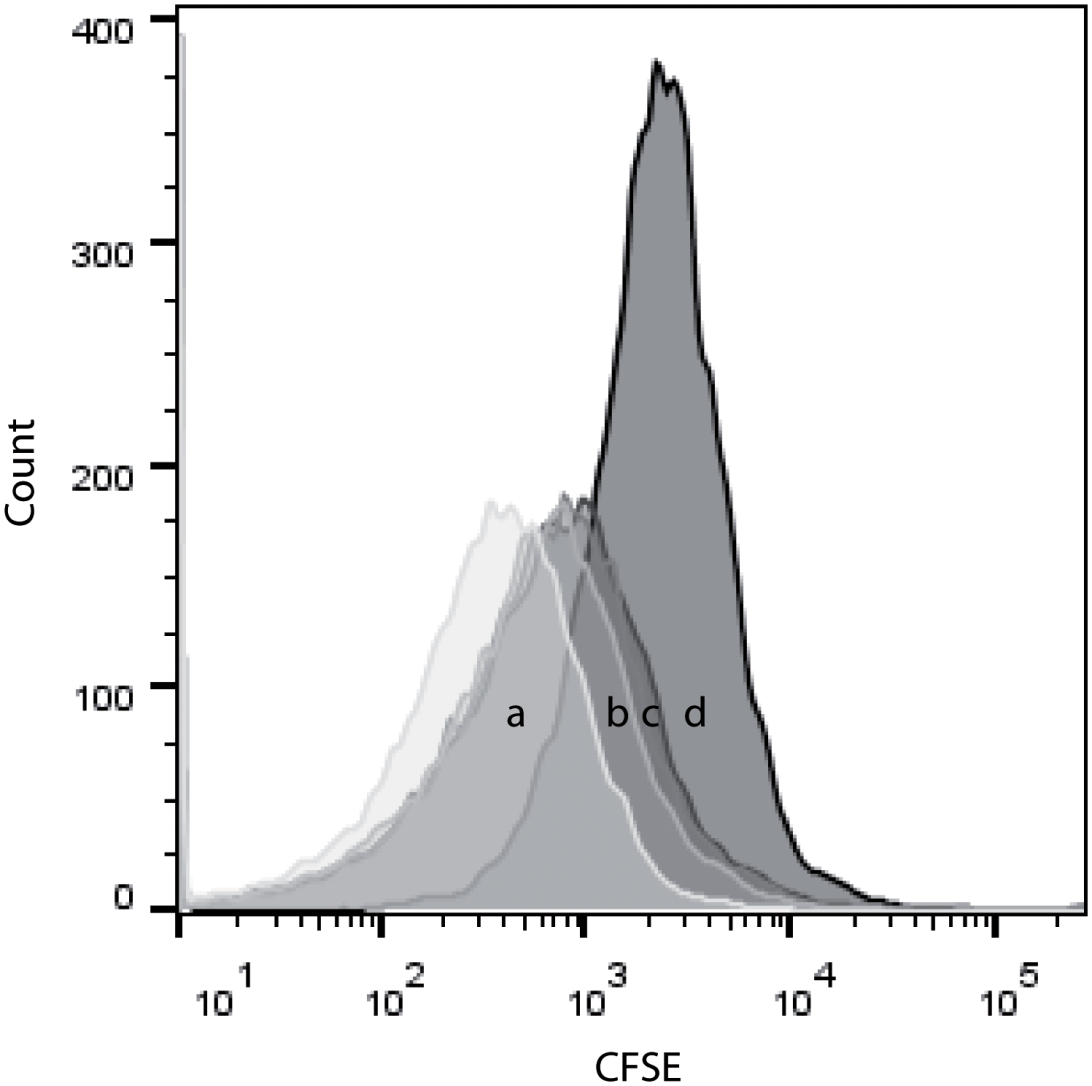
Histograms showing the fluorescence of CFSE stained T-cells. (a) At day 3, the fluorescence of responders/stimulators at 1:1 ratio significantly shifted to the left when compared to the (d) fluorescence of responders alone (*p* = 0.02564). Effect was ratio dependent with 1:2 (b), 1:4 (c) bDCs:responders showing less decrease in fluorescence over three days than 1:1(a). Data presented are representative of three biological replicate data sets.

The defining role of antigen presenting cells, and especially of dendritic cells, is to stimulate naïve lymphocytes and trigger their proliferation. To determine if enriched cells were able to trigger T-lymphocyte proliferation, a proliferation assay, was performed. CFSE fluorescence of cells is typically reduced each generation by 50%, making the distinction of daughter cells possible. To determine background cell division, responder cells were incubated alone. After three days, the CFSE fluorescence of responder cells alone decreased slightly, but not significantly (*p* > 0.05) when compared to fluorescence at day 0 (data not shown). However, when incubated for three days with bDCs (Fig 7), the CFSE fluorescence of T-cells decreased significantly in comparison to the fluorescence of responders alone after the same time. Decrease in fluorescence occurred in a dose-dependent manner, with maximum decrease in CFSE fluorescence achieved with a bDC:responder ratio of 1:1 (Fig 7). The CFSE fluorescence of responders and stimulators combined at day 3 was also significantly lower than the CFSE fluorescence of responders and stimulators at day 0, whereas the CFSE fluorescence of responders alone after three days was not significantly different from the CFSE fluorescence at day 0 (data not shown).

## Discussion

A subset of barramundi cells derived from head kidney and spleen monocytes expressed DC-SCRIPT and exhibited morphological and functional characteristics of mammalian dendritic cells (mDCs). DC-SCRIPT is a transcriptional regulator of the Zinc finger family that is preferentially expressed in all subsets of human DCs [16]. The putative DC-SCRIPT protein identified in this study was highly similar to human and murine DC-SCRIPT, with a similar structure and highly conserved zinc fingers. A homologue has been previously identified in the fugu genome *in silico*, but this study represents the first identification of DC-SCRIPT in putative teleost DCs. DC-SCRIPT did not appear to be expressed in other blood cells in mixed cell cultures from barramundi, similar to findings in human blood cells [16, 32], making teleost DC-SCRIPT a potentially useful molecular marker for DCs in fish. In fluorescence microscopy, bDCs were labelled with fluorescent mRNA probes directed against DC-SCRIPT and were also positive for MHCII using anti-zebrafish MHCIIa. The anti-MHCII antibody employed is a commercial polyclonal antibody raised in rabbits against a peptide comprising the transmembrane MHCIIa and MHCIIa-Ig superfamily domains from zebrafish (NP_571565.1) and was chosen as likely to be cross reactive with barramundi MHCII based on protein homology by BLAST with the barramundi MHCIIa Ig superfamily region (derived from partial gDNA sequence), and that of fish from several orders including other cyprinids (*C*. *carpio*), salmonids (*S*. *salar*) and perciforms (*O*. *niloticus*, *M*. *zebra*). Epitope mapping revealed highly conserved B-cell epitopes in both the MHCIIa domain and the Ig superfamily domain of the zebrafish peptide (S2 Fig). Moreover co-labelling with specific anti-barramundi IgM and the anti-zebrafish MHCII in barramundi B-cells was evident (S2 Fig).

DC-SCRIPT expression determined by qRT-PCR indicated that bDCs are present in spleen and HK at the beginning of an infection and that DC-SCRIPT expression in these organs was increased, possibly via cytokine signalling from primary responder cells at the site of injection, within 6 h post injection. As the innate and adaptive immune response develops, DC-SCRIPT expression decreases in spleen, possibly indicative that bDCs have migrated from the spleen towards the site of infection. At 72 h post infection, DC-SCRIPT expression was also reduced in HK, also suggesting a migration from the HK. However, after 7 days, DC-SCRIPT was again elevated in spleen but not in HK, which suggests that bDCs have migrated back to the spleen, possibly for maturation and antigen presentation. These kinetics appear to be consistent with previous studies on appearance of antibody secreting cells in tissues in European sea bass [33]. This is important against a background of fish vaccination for sustainable disease control in aquaculture. Previously we have demonstrated that a number of antigens elicit poor antibody responses in vaccinated barramundi [34]. In human DCs, DC-SCRIPT appears to control glucocorticoid (GC) suppression of antigen presentation by binding to the GC receptors [35]. It will be interesting to determine how DC-SCRIPT is expressed in fish DCs post-vaccination with vaccines that elicit both strong and poor antibody responses. Novel adjuvants that enhance DC-SCRIPT expression may be a way of promoting presentation of critical but poorly immunogenic antigens.

Barramundi cells, cultured from spleen and HK, were able to differentiate into highly motile, irregular shaped cells with low buoyancy, similar to mDCs and rainbow trout dendritic cells (tDCs) [15, 29–31]. These cells could also arise in HK and spleen cultures individually as well as in blood leucocyte cultures, although no functional studies have been performed yet on these culture and blood-derived cells. As observed in cultures from rainbow trout head kidney, anterior trunk kidney, spleen, and mouse spleen, barramundi DCs seemed to be generated from hematopoietic tissues without addition of an exogenous growth factor source [15, 36]. This could indicate the presence of endogenous growth factors in primary barramundi spleen and HK cultures. This theory is supported by the quick de-differentiation of these cells when cultured in fresh supplemented medium (L-15/10%FBS/1%PS) after enrichment and by the slow/non differentiation of spleen and HK cultures into bDCs when the medium was changed every day instead of every 4–5 days (data not shown).

In addition to identifying DCs by their morphological properties, the current study characterised their function as putative APCs, using a proliferation assay. Classically, in mixed leucocyte reactions, CFSE histograms present several peaks, each representing a unique cell division. In this study, however, CFSE histograms only presented one peak, which shifted to the left when T-cells were exposed to bDCs for 3 days. This dissimilarity with other studies is likely explained by the method used to isolate the T-cells subsequently used in the proliferation assays. As no T-cell markers are available in barramundi to enable enrichment of T-cells from mixed leucocyte populations we used the E-rosette method to obtain a homogeneous population of viable T-cells. E-rosetting is a method that relies on the binding of T-cells CD2 by a LFA-3 homologue present at the surface of sheep red blood cells, forming “rosettes” [37]. This method has been extensively used in humans and mice until the commercialisation of magnetised beads, and results in high yields of viable active T-cells [27, 38]. More recently, the E-rosette method has also been used to isolate T-cells from mud catfish peripheral blood, indicating that this technique is efficient not only in mammals but also in fish [39]. In contrast, many previous studies obtain responder cell populations by removing B-cells from mixed leucocyte cultures by FACS or magnetic beads sorting, resulting in a mixed leucocyte population. The presence of cells other than T-cells in the MLR can account for the presence of several well-defined CFSE peaks through differing cell division rates. In contrast in this study, bDCs were able to stimulate proliferation of the whole T-cell population, resulting in the CFSE fluorescence decreasing by half after 3 days compared to T-cells alone, or bDCs plus T-cells at day 0. This suggests that isolated cells are functionally equivalent to mDCs.

Another key characteristic of APCs that is critical to antigen presentation is their ability to sense non-self substances and migrate towards them by chemotaxis [7]. Barramundi DCs were attracted to TLR-ligands and whole bacteria and were able to phagocytose particulates including bacteria and opsonised beads. Barramundi DCs did not migrate towards LPS, which suggests that bDCs were unable to recognise and bind TLR-4 ligands in contrast to mammalian DCs. TLRs are proteins capable of recognising a range of exogenous and endogenous ligands, including pathogen associated molecular patterns (PAMPs). In mammals, the lipopolysaccharide binding protein (LBP), located in serum, mediates the interaction between LPS on bacteria surface and the glycoprotein CD14 on phagocytic cells [40]. Additionally, TLR-4 must be coupled with the myeloid differentiation protein 2 (MD-2) to functionally interact with LPS [41]. The lack of significant attraction of bDCs to LPS in the present study is likely due to a lack of LBP in the culture system or to a lack of CD14/MD-2 on bDCs surface: to date, CD14 and MD-2 are absent from all fish genomes sequenced [42]. It may also be that higher concentrations of LPS than used in the present study are required to activate fish immune cells [43]. The lack of reaction to LPS by bDCs could present an issue both for future vaccine design and for *in vitro* studies on *L*. *calcarifer*, as TLR-4 ligands are frequently used to stimulate DCs for *in vitro* studies in mammals [44, 45]. Further work is warranted to determine whether barramundi do indeed have intermediate LBP that could be included in the assays system to mediate interaction with TLR-4.

Finally, the characterisation of DCs in *L*. *calcarifer* provides a baseline for future vaccine design against *S*. *iniae* and other pathogens. Vaccination in fish has experienced some failures due to rapid evolution of key immunogens and consequent vaccine escape [46, 47]. For example, killed whole-cell vaccines against *S*. *iniae*, which is a major pathogen of warm water fish, are often compromised by rapid-evolution of novel serotypes [47, 48]. Vaccines against *S*. *iniae* are effective against the specific serotype in the vaccine, with the fish adaptive immune system responding to the capsular polysaccharide (CPS), which is immunodominant [47, 49, 50]. However, the CPS is highly variable and new CPS variants of *S*. *iniae* rapidly emerge, leading to reinfection and disease outbreaks in previously vaccinated fish [47, 50]. Moreover, although there are conserved critical virulence factors in *S*. *iniae*, such as the surface-expressed M-protein, the immune system does not seem to develop an effective response against them [25]. Enhancing antigen presentation of non-immunodominant conserved virulence factors is therefore a critical step in the development of broadly cross-protective vaccines against these highly variable and rapidly mutating strains. The main cells that need to be targeted during vaccination are APCs, and in particular dendritic cells that will activate pathways leading to immune memory [3, 8]. The capacity to evaluate multiple antigens across a single population of APCs *in vitro*, rather than vaccinating cohorts of fish with each strain or antigen has immense value in terms of rapid high throughput screening of putative antigens. *In vitro* experiments also have great merit in terms of animal welfare as these assays greatly reduce animal numbers required and refine (reduce the stress of) the procedures conducted on animals, in line with the ethical requirements of the three R’s (Replacement, Reduction and Refinement).

The identification of a molecular marker for dendritic cells in barramundi provides a potentially useful tool for new flow-cytometric investigations of antigen presentation in fish. Work is ongoing to determine the function of DC-SCRIPT in bDCS and elucidate its role in antigen presentation.

## Supporting Information

S1 Fig E-rosettes. (a) Inverted light microscopy of rosette formations. (b-c) Rosettes stained with Hemacolour. Scale bars represent 50 μm in (a) and 20 μm in (b) and (c). In each case, putative T-cells are surrounded by sheep erythrocytes.
(PDF)

S2 Fig Rabbit Anti-zebrafish MHCIIa (Sapphire Bioscience). A) Synthesised peptide amino acid sequence, showing MHCIIa antigen (green) and MHCII-Ig superfamily domains (purple). Alignment: A subsection of exon from *L*. *calcarifer* partial gDNA sequence is highly conserved across *D*. *rerio*, *L*. *calcarifer*, *M*. *zebra* and *O*. *niloticus*. A B-cell epitope region is indicated (shaded, bold) showing very high conservation across several fish orders (Standard ClustalW2 notation to indicate similarity/identity). B) Co-staining of B-lymphocyte from *L*. *calcarifer* with rabbit anti-zebrafish MHCIIa antibody (green), sheep anti-barramundi IgM (blue).
(PDF)
