# Supplementary material for: Identification of Barramundi (Lates calcarifer) DC-SCRIPT, a Specific Molecular Marker for Dendritic Cells in Fish
Source: PLoS One. 2015 Jul 14;10(7):e0132687. doi: 10.1371/journal.pone.0132687 (PMC4501824; doi:10.1371/journal.pone.0132687)
Supplement: S1 Fig — (a) Inverted light microscopy of rosette formations. (b-c) Rosettes stained with Hemacolour. Scale bars represent 50 μm in (a) and 20 μm in (b) and (c). In each case, putative T-cells are surrounded by sheep erythrocytes. (PDF) [file pone.0132687.s001.pdf]

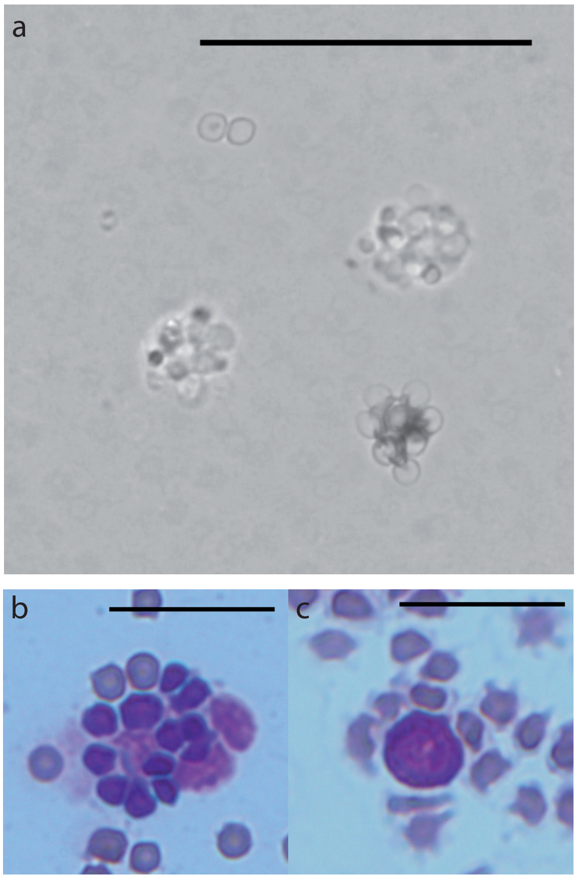

S1 Fig. E-rosettes. (a) Inverted light microscopy of rosette formations. (b-c) Rosettes stained with Hemacolour. Scale bars represent 50 μm in (a) and 20 μm in (b) and (c).
