# Supplementary material for: Identification of Barramundi (Lates calcarifer) DC-SCRIPT, a Specific Molecular Marker for Dendritic Cells in Fish
Source: PLoS One. 2015 Jul 14;10(7):e0132687. doi: 10.1371/journal.pone.0132687 (PMC4501824; doi:10.1371/journal.pone.0132687)
Supplement: S2 Fig — A) Synthesised peptide amino acid sequence, showing MHCIIa antigen (green) and MHCII-Ig superfamily domains (purple). Alignment: A subsection of exon from L. calcarifer partial gDNA sequence is highly conserved across D. rerio, L. calcarifer, M. zebra and O. niloticus. A B-cell epitope region is indicated (shaded, bold) showing very high conservation across several fish orders (Standard ClustalW2 notation to indicate similarity/identity). B) Co-staining of B-lymphocyte from L. calcarifer with rabbit anti-zebrafish MHCIIa antibody (green), sheep anti-barramundi IgM (blue). (PDF) [file pone.0132687.s002.pdf]

a

NP_571565.1| major histocompatibility complex class II integral membrane alpha chain precursor [Danio rerio]

MFFLALRKMEVYVFILTLSVFVSSEVNVVHEDIVMDGCSDTEKEYISVLDGEEMYHT
DFSGKRGEMTLPDFADPFTYPGTYEQSLADYETCKHNLDVAAKAYKSPLEKLDPPQT
SIYSRDDVQPDIENKLICHVTGFFPPPVRVSWTKNNEIVTEGMSVSQYRPNNDGTYN
IFSTLRFTPVEGDIYSCSVNHKTLEQPQTKAWEVEVAMPSVGPAVFCGVGLFLGLLG
VAAGTFFLIKGNNCN

D. rerio	LICHVTGF**FPPPVRVSWT**KNNEIVTEGMSVSQYRPNNDG
L. calcarifer	LICHVSGF**YPAPVNVSWT**KNEQKVTEGTSINVPFPLKDG
M. zebra	LICHVTGF**YPAPVNVSWT**KNGQKVT-GSSINVPYPNKDG
O. niloticus	LICHVTGF**YPAPVNVSWT**KNQQKVT-GSTINVPYPNKDG
	*****:**:*.**.****** : **** ::. * :**

b

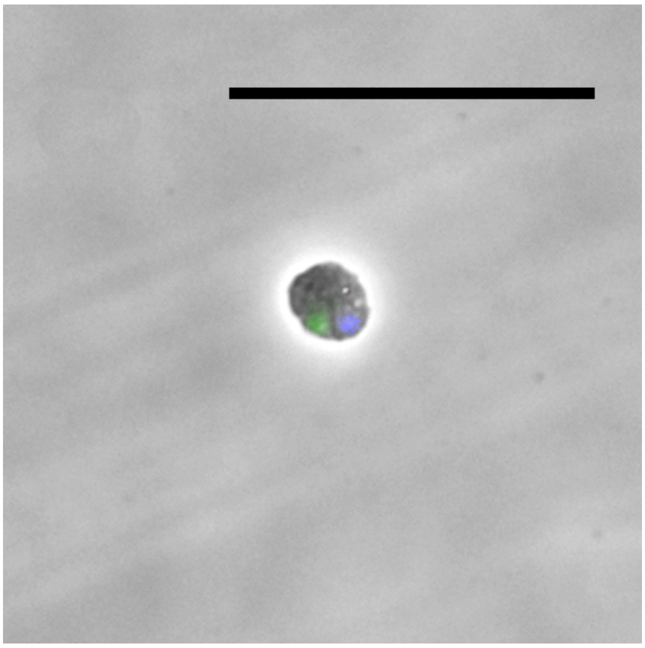

S2 Fig. Rabbit Anti-zebrafish MHCIIa (Sapphire Bioscience)

a) Synthesised peptide amino acid sequence, showing MHCIIa antigen (green) and MHCII-Ig superfamily domains (purple). Alignment: A subsection of exon from *L. calcarifer* partial gDNA sequence is highly conserved across *D. rerio, L. calcarifer, M. zebra* and *O. niloticus*. A B-cell epitope region is indicated (shaded, bold) showing very high conservation across several fish orders (Standard ClustalW2 notation to indicate similarity/identity). b) Co-staining of B-lymphocyte from *L. calcarifer* with rabbit anti-zebrafish MHCIIa antibody (green), sheep anti-barramundi IgM (blue)
